# Network Properties for Ranking Predicted miRNA Targets in Breast Cancer

**DOI:** 10.1155/2009/182689

**Published:** 2010-03-07

**Authors:** Jörg Linde, Björn Olsson, Zelmina Lubovac

**Affiliations:** ^1^Leibniz-Institute for Natural Product Research and Infection Biology, Hans-Knoell-Institute, Beutenbergstraße 11A, 07745 Jena, Germany; ^2^School of Life Sciences, Systems Biology Research Centre, University of Skövde, P.O. Box 408, 54128 Skövde, Sweden

## Abstract

MicroRNAs control the expression of their target genes by translational repression and transcriptional cleavage. They are involved in various biological processes including development and progression of cancer. To uncover the biological role of miRNAs it is important to identify their target genes. The small number of experimentally validated target genes makes computer prediction methods very important. However, state-of-the-art prediction tools result in a great number of putative targets with an unpredictable number of false positives. In this paper, we propose and evaluate two approaches for ranking the biological relevance of putative targets of miRNAs which are associated with breast cancer.

## 1. Introduction

It has been uncovered that microRNAs (miRNAs) play an important role in several cellular processes, such as development, cell proliferation, differentiation, and apoptosis, which are crucial in a number of diseases, including cancer [1, 2]. The prediction of their target genes is an important step towards uncovering the role of miRNAs. However, state-of-the-art prediction tools result in a large number of putative target genes, many of which may be false positives. 

 The small number of experimentally validated target genes [3] makes it difficult to determine the number of false hits [4]. In addition, even if a highly specific prediction algorithm could be envisaged, we cannot be sure which target gene is relevant in a biological sense. For example, a predicted target might only be seen as relevant if the putative target gene and the miRNA gene are temporarily and locally coexpressed. 

 This paper investigates different properties of putative miRNA target genes that may be used to rank these genes and suggest their biological relevance. The list of putative miRNA targets originates from the study by Iorio et al. [5], where 29 significantly deregulated miRNAs in human breast cancer were identified. Of these 29 miRNAs, our study focuses on the five that are most consistently differentially expressed between normal and breast cancer tissues. 

 Most existing prediction tools [6–8] use sequence-based or three-dimensional complex analysis to find target genes and evolutionary sequence conservation to reduce the number of false positives. We attempt to complement these approaches by using more biological background information, in order to find the most relevant targets among these putative target genes. As we focus on the targets of the miRNAs that are involved in breast cancer, we expect these targets to play important roles in cancer, and thereby act as interesting candidates as cancer biomarkers. The list of target genes generated by our approaches provides a starting point for further experimental validation. It has been suggested that miRNAs and their targets might be used as both biomarkers and drug targets [1]. 

 In a recent study, Liang and Li [9] investigated the role of miRNAs in regulation of protein-protein interaction networks (PPINs). MiRNAs preferentially regulate proteins which have a higher than average number of interacting partners in the network. Node degree in a human PPIN is positively correlated with the number of miRNA target-site types. It has also been shown that proteins having many interacting neighbours normally take part in more biological processes [10]. Therefore, it is reasonable to expect that a protein that has more interacting protein partners is regulated by more transcription factors and more miRNAs. The use of network topological properties, such as node degree, to characterise cancer-related proteins is also supported by the finding that cancer-related proteins have larger mean degree value than the mean value of the human PPIN [11]. 

 Hence, we characterise these properties of the nodes in the network corresponding to putative target genes and compare them to the mean values of the network. In this way we investigate different ways of ranking the putative target genes according to their roles within the PPIN. To evaluate different approaches we use a data set of experimentally validated miRNA target genes [3] and a data set of genes which are known to be involved in breast cancer [12]. We are able to show that the network properties used in this study contribute differentially to the ranking approach, and furthermore that our ranking approach is capable to discriminate between genes involved in breast cancer and genes which seem not to be involved.

## 2. Materials and Methods

### 2.1. Putative Target Genes of Deregulated miRNAs in Breast Cancer

Iorio et al. [5] identified 29 significantly deregulated miRNAs in human breast cancer. The five most consistently differentially expressed miRNAs are miR10b, miR125b, miR145, miR21, and miR155. To generate a list of putative targets the authors used three tools, namely, miRanda [13], Pictar [14], and TargetScan [15]. For more reliable results, only targets predicted by at least two of the tools were included in the result. Thus they generated a list of 719 putative target genes for these five miRNAs. (the list is available as supplementary material in Iorio et al. [5] ) We had to exclude some of these genes for either of the following reasons: (1) they are from the mouse or rat genome and no human homologue is known, (2) they are not included in the human PPIN. Out of the 719 putative target genes, 465 passed the exclusion criteria.

### 2.2. Experimentally Validated miRNA Target Genes

We used TarBase [3] to access experimentally validated miRNA targets in order to compare them to putative targets. In March 2008 TarBase contained 461 validated human miRNA target sites from 418 different genes. Out of those 418 genes, 213 passed the exclusion criteria (see above). For the five most consistently differentially expressed miRNAs identified by Iorio et al. [5], the TarBase search resulted in 19 experimentally validated target genes. The list of putative target genes generated by Iorio et al. [5] does not include any of these validated target genes for these five miRNAs. However, out of the 418 validated human target genes, 22 can be found in the list of Iorio et al. [5]. Note that these are targets of other miRNAs than those five most consistently deregulated.

### 2.3. Protein-Protein Interaction Network

The human PPIN was downloaded from the Human Protein Reference Database (accessed in September 2008) [16] which contains manually curated protein-protein interactions between 9162 proteins. As identifiers we used Gene Symbols. Network visualization and analysis tool Cytoscape [17] was used to find the putative target genes for deregulated miRNAs in breast cancer within PPIN.

### 2.4. Breast Cancer Genes

We downloaded genes which are known to be involved in breast cancer from the Breast Cancer Gene Database (accessed in April 2008) [12]. The database is manually curated and the data is extracted from literature search. We downloaded a list of 72 genes which we use to evaluate our results. However, we could not use all of them because they were not included in the human PPIN, nor annotated by the GO categories. Out of those 72 genes, 27 are located in the human PPIN but only one is among the 465 putative target genes we used for the PPIN analysis.

### 2.5. Network Measures

The following chapter describes network properties used in this study. The igraph package [18] of the statistical language R [19] was used to calculate each network property value for each node within the PPIN. Mean values were calculated for the following sets of nodes: the whole PPIN, the putative miRNA targets, and the validated miRNA targets. The Mann-Whitney-Wilcoxon test was used to test for significant differences in the distributions of the different data sets for all four network properties.

### 2.6. Degree

The degree value of a vertex is the number of directed neighbours of the vertex.

### 2.7. Betweenness Centrality

Betweenness centrality has been applied in the context of social networks, to measure the centrality and influence of a person or a group [20]. The betweenness centrality of a node *v* was originally defined by Freeman [20] as the number of shortest paths (also called *geodesics*) between other nodes that pass through *v* and it is given by

(1) $$C_{b}\left(v\right)=\underset{i,j\in V:\;\;i\neq j,i\neq v,j\neq v}{\sum }\frac{g_{ivj}}{g_{ij}},$$

where *g*_*ivj*_ is the number of shortest paths linking *i* and *j* that contain *v*, and *g*_*ij*_ is the total number of shortest paths between *i* and *j*. High-betweenness nodes occur on large number of nonredundant shortest paths between other nodes. If a node with high betweenness centrality is removed, it may disconnect different parts of the network completely. Thus, such nodes may be thought of as potential bridges between modules in network and have most influence on the information transfer.

### 2.8. Closeness Centrality

Another centrality measure is closeness centrality which is defined as the mean shortest path length between a vertex and all other vertices reachable from it. It is defined as

(2) $$\text{Closeness}\left(v\right)=\frac{\sum_{t\in V-v}d\left(v,t\right)}{n-1},$$

where *d*(*v*, *t*) is the shortest distance between *v* and *t*, and *n* is the number of reachable vertices from *v*. Vertices which have mean short distances to other ones in the network have greater closeness centrality values.

### 2.9. Clustering Coefficient

The clustering coefficient value is the fraction of connected neighbours of a vertex *i* divided by the number of all possible connections of the neighbours of *i* [21]. In other words it measures how close a vertex and its neighbours are to be totally connected and thus form a “clique.” Many cliques contribute to a small world network because you can reach every vertex on a short path [21]. The clustering coefficient of an undirected graph is defined as two times the number of edges between the neighbours divided by the number of all possible edges between the neighbours

(3) $$C_{c}\left(v\right)=\frac{2N\left(v\right)}{n\left(n-1\right)},$$

where *N*(*v*) is the number of edges between the neighbours of *v*, and *n* is the number of neighbours of *v*.

### 2.10. Ranking Approaches

**Figure 1:**
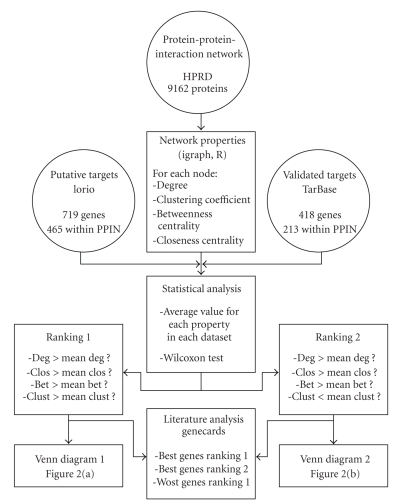
Overview of the ranking approach.

In this study we applied a simple ranking approach to access the biological relevance of a putative miRNA target gene. We compared all four network property values of a putative target gene to the mean values within the whole PPIN. For degree, betweenness centrality and closeness centrality greater mean values were found in both the validated and putative target genes compared to the whole PPIN. For this reason we checked for each putative target if it has a greater value than the mean of the PPIN for these three properties. For clustering coefficient the putative targets have a greater mean value than the mean of the PPIN while the putative targets have a smaller one. For this reason we test both a “greater than” and a “smaller than” criteria for this network property. The ranking approach and data sets used in this study are summarised in Figure 1.

## 3. Results

### 3.1. Ranking according to PPIN Properties

In order to investigate potential methods to rank putative target genes with the help of PPIN properties we first characterised a set of properties for the whole PPIN, and the putative and validated target sets (see Section 2). For each data set we calculated the mean degree *k*, betweenness centrality (*C_b_*), closeness centrality (*Closeness*), and clustering coefficient values (*C_c_*) (see Table 1). 

 Both the validated and the putative target data sets are characterised by greater mean degree values than PPIN, which suggests that this property is the most consistently different from the whole human PPIN. A similar result was obtained for betweenness centrality, where the networks of validated and putative targets show higher betweenness centrality than PPIN, and the corresponding value for validated targets is significantly higher than for the putative targets. The data set of validated targets is characterised by the highest mean betweenness centrality which suggests the presence of hubs among the validated targets to a greater extent compared to other networks.

The mean closeness centrality values do not differ significantly between any of the datasets. The mean clustering coefficient is slightly smaller for the validated target sets but greater for the putative ones, compared to the mean value of the whole PPIN. In contrast, the mean degree is greater for the validated targets than for the putative targets. 

 This result is highly interesting and adds additional evidence to what has been proposed by Liang and Li [9]. In short, they suggested that different types of hub proteins have different miRNA targeting propensity, that is, intramodular hubs (usually termed modules) do not tend to contain miRNA target sites, whereas intermodular hubs often contain several miRNA target sites. Intermodular hubs, which are often involved in a variety of cellular processes, such as transcription regulation and proliferation, are hereby suggested by Liang and Li [9] to play a more important role for miRNA regulation than their counterparts [9]. In this study we were able to show that also betweenness centrality may be useful as a complement to existing properties, as validated target genes show significantly higher values for this property. 

 To test the significance of these results the Mann-Whitney-Wilcoxon (MWW) signed rank test [22] was used for all four properties and all three data sets in a pairwise way. The result is a *P*-value which is the probability of observing these differences between mean values by chance. Pairwise results are shown in Table 2. For both the mean degree and betweenness centrality values, all pairwise differences are significant. The closeness centrality values differ significantly between the whole PPIN and both the validated and putative targets, while the corresponding value for the putative targets and validated targets is not significant. The differences for the mean clustering coefficient values are only significant between the whole PPIN and the data set of putative targets.

**Figure 2:**
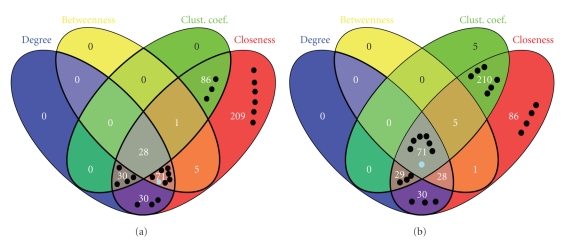
Venn diagram showing overlapping of network properties. (a) A criterion “greater than” was used for clustering coefficient. (b) A criterion “smaller than” was used for clustering coefficient. For example, the 28 in the grey section of (a) indicates that there are 28 putative targets which have a greater network property value than the mean value of the whole PPIN for all four properties used and are thus best ranked.

In the next step we studied which network properties or combinations of network properties contribute most to the ranking. In order to perform that, we calculated how many vertices have greater/smaller values (depending on how the mean property of validated and putative targets differs from the mean property of the whole PPIN, see Materials and methods) than the mean values for all four network properties and all 12 combinations of them. For easier interpretation this is visualised with the help of Venn Diagrams. Such diagrams are shown for both the “greater than” criterion (Figure 2(a)) and “smaller than” criterion (Figure 2(b)) regarding the clustering coefficient (see Section 2).

For further evaluation we located the 22 validated miRNA targets and the one known breast cancer gene which are members of the 460 putative targets in the Venn diagrams. Figure 2 shows in which subsets these proteins are located. The diagrams show that the putative targets are not members of a distinct subset of a combination of network properties. Rather, they can be found in different kinds of subsets. Generally, however, fewer putative targets can be found when more properties are combined. 

 In Figure 2(a) there are 14 validated out of 159 putative targets within the sets of degree, while in Figure 2(b) there are 13 out of 158. Thus degree maximizes the ratio of maximum validated targets and minimum putative targets. 

 All (Figure 2(a)) or all but five (Figure 2(b)) putative targets are located in the subset generated based on closeness centrality. Again, closeness centrality does not seem to contribute a lot to the ranking, using our approach.

### 3.2. Literature Analysis

Literature research was done for the following sets of genes

1. Ten of the best ranked genes using the “smaller than” criterion for clustering coefficient values.
2. Ten of the best ranked genes using the “greater than” criterion for clustering coefficient values.
3. Ten of the worst ranked using the “smaller than” criterion for clustering coefficient values.

We used the GeneCards [23] Database to gain information about genes and diseases in which they are involved. 

 For every gene the database shows a table which summarises results of the literature research. For 92 diseases it is given a score of the relevance of the disease to this gene which is based on the analysis of co-occurrences of the gene and the disease in Medline articles. If there exist cancer-related diseases among the ten highest scored diseases a maximum of three of them is shown in Table 3 (column 2). Furthermore, the number of articles in which the gene and the words “breast cancer” occur together is shown (Table 3, column 3). To assess the statistical significance of this finding the Novoseek score [23] of the relevance of the disease to this gene is given. (Table 3, column 4) This score compares the number of documents in which the gene name and the words “breast cancer” occur together with the number of documents where both appear independently based on a hypergeometric distribution. The more co-occurrences are found in relation to the number of expected, the more unlikely it is that the gene and the disease occur together by chance. The absolute numbers of the score are not meaningful, but they help to compare the different ranking results. 

Table 3 summarises results of the literature research. The first and the second parts of the table show results for the top ranked genes for the “smaller than” and “greater than” criterion (top ten ranks), respectively. The third part shows results for the ten lowest ranked genes. The putative targets in the first part are mostly involved in functions such as transcription regulation or signal transduction. For almost all of them there are cancer-related articles among the top ten articles of diseases in which the genes are mentioned. Moreover breast cancer is often among the top three of these types of cancer-related articles. Especially for the genes SHC1 and IRS1 there were 40 and 96 relevant articles found, respectively. This might imply that this approach is capable of finding breast cancer related genes. Only for the protein NEDD9 there was no cancer related article found. However, only for five of the ten best ranked putative targets (using the “smaller than” criterion) were any cancer related article among the top ten disease related articles. 

 No breast cancer related article was found for any of the ten lowest ranked genes, and for only one of them was a cancer related article among the ten top-ranked articles. This shows that our approach is able to rank genes which are involved in breast cancer highly while genes which are not known to be involved in breast cancer are lowly ranked. 

 The NovoSeek score seems to be quite conservative in general. However, with the help of the first ranking approach, genes were identified whose occurrence in breast cancer related articles does not happen by chance. This is not the case for the second ranking approach and the worst ranked genes, respectively.

## 4. Conclusions and Discussion

In this study we used network properties of putative miRNA target genes in an attempt to rank them according to these properties and to identify the biologically plausible targets. We characterised a set of properties within the whole human PPIN and compared it to the properties of sets of validated and putative targets. The mean network property values of both the putative and the validated targets differ significantly from those of the whole human PPIN. This difference should be useful to rank the putative target genes according to relevance. 

 The mean degree values differ most consistently between the whole human PPIN and all other data sets. Furthermore, using the “greater than” criterion for degree leads to the greatest ratio between the number of validated targets in the data set and the total number of putative targets. This suggests that using degree for ranking may result in high specificity. 

 Another measurement producing data sets that differ consistently and significantly from the whole human PPIN is closeness centrality. The mean values suggest that the putative and validated targets have greater mean closeness centrality values. However, only a very small number of vertices have smaller closeness centrality values which makes it very difficult to rank based on a “greater than” criterion for the mean value. 

 The mean clustering coefficient value is greater for the validated targets but smaller for the putative ones. This is the largest difference between the putative and the validated targets and might be used for ranking purposes. However, the number of putative targets having a smaller clustering coefficient than the mean one is high, which might lead to low specificity. Using the “smaller than” criterion for the clustering coefficient in combination with “greater than” for degree also leads to a great number of validated targets but a small number of putative targets. The literature search suggests that using the proteins that have lower clustering coefficients than the mean value results in target genes associated to more cancer related articles than the best ranked putative targets having larger clustering coefficient than the mean. 

 Although we cannot draw a general conclusion that the validated targets are members of a distinct subset, there is a tendency that they are found more often in the subsets of combined properties than in sets where only one criterion based on one property is satisfied. In both cases there are a large number of both putative and validated targets within the subsets generated based on closeness centrality values. 

 State-of-the-art miRNA target gene prediction tools use sequence-based or three-dimensional complex analysis to find target genes and evolutionary sequence conservation to reduce the number of false positives. However, the number of false positives is still high and difficult to calculate. This study attempts to complement these tools by applying a ranking approach based on network properties to access the biological most relevant putative miRNA target genes. For each putative target we test if a set of network properties is greater than the mean value of the whole human PPIN (also smaller than in the case of clustering coefficient). 

 This methodology is applied to putative targets of miRNAs in breast cancer. Even though a simple “Boolean” ranking approach is used, we are able to rank genes involved in breast cancer highly while genes with a low rank do not seem to play a role in breast cancer. Future contributions could incorporate the distributions of the network properties and thus study if the absolute value of the difference to the mean value of a network property helps more for ranking. Furthermore, we are sure that our approach could be more generalized. 

 In a recent work by Yuan et al. [24], an attempt has been made to demonstrate the functional connection (coregulation) between clustered miRNAs and the target proteins that are closely located in PPIN [24]. Hence, the functionality of miRNAs is analysed according to the topological features of their target proteins. It would be highly interesting to apply the similar approach to validated and putative targets of miRNAs in breast cancer, to investigate if the hypothesis proposed in [24] can be validated in cancer-related network, and also analyse it in relation to other topological aspects, besides connection and proximity. This would be valuable to guide the experiments towards the regions of PPINs likely to modulate cancer-related processes, which may be important for future work with revealing putative therapeutics targets in cancer.

## Figures and Tables

**Table 1:** Mean values of network properties. The table summarises the values of different network properties for three datasets: the whole PPIN, the putative targets, and the validated targets. Abbreviations: *k*: degree, *C*_*b*_: betweenness centrality, Closeness: closeness centrality, *C*_*C*_: clustering coefficient.

Property/network	PPIN	Putative	Validated
*k*	7.48	9.67	13.51
*C_b_*	14185	18164	34389
*Closeness*	0.0038	0.0039	0.0039
*C_c_*	0.1047	0.1210	0.0825

**Table 2:** Significance test for network properties. The *P*-value indicates the probability of observing the different mean values by chance. All tests are two tailed. Bold values indicate non significant results (*P* > .05). Abbreviations, *k*: degree, *C*_*B*_: betweenness centrality, *C*_*C*_: clustering coefficient, Closeness: closeness centrality.

Samples	*k*	*C_B_*	*C_C_*	*Closeness*
PPIN versus putative	9.4 10^−8^	0.0001	3.0 10^−6^	1.7 10^−10^
PPIN versus validated	3.0 10^−8^	2.7 10^−8^	**0.9857**	2.2 10^−16^
validated versus putative	0.0470	0.0040	**0.1523**	**0.1754**

**Table 3:** Literature research results for ranking according to PPIN properties. The first part shows information for the ten best ranked putative targets using the “greater than” criterion for clustering coefficient values and the second part the same for using the “smaller than” criterion. The last part shows results for the ten worst ranked genes.

Gene	Cancer types	Articles	Score
SHC1	Men 2a, breast cancer, men 3	42	38.16
CRK	Myeloid leukemia chronic	1	0
IRS1	Breast cancer	102	40.49
CEBPB	Choriocarcinoma, tumors, leukemia	2	0
SOCS1	Carcinoma, colorectal cancer, tumors	7	0
NEDD9	Cancer, tumor	0	0
NCOA6	Cancer	0	0
NRIP1	Carcinoma embryonal, breast cancer	8	46.76
ETS1	Leukemia, tumors, leukemia t-cell	27	23.86
BTRC	Tumors, cancer, colorectal cancer	6	0
YES1	Colon cancer, mammary tumor, colon	0	0
	carcinoma		
RPS6KA3	—	0	0
RHOQ	—	0	0
GJA1	Carcinoma giant cell, tumors	38	0
MAP3K10	—	0	0
MAP3K11	Tumors	0	0
KPNA1	—	0	0
ACVR2A	Colon cancer, pancreatic cancer, tumors	0	0
SDC1	Carcinoma, breast cancer	1	0
RGS7	—	0	0
SMNDC1	—	0	0
PURB	—	0	0
GGTL3	—	0	0
PRPF4B	—	0	0
SSFA2	—	0	0
POMT2	—	0	0
HCN4	—	0	0
GRHL1	—	0	0
ABCG1	—	0	0
MAT2A	Carcinoma, cancer, tumor	0	0
